# Ecologically Acceptable usage of Derivatives of Essential Oil of Sweet Basil, *Ocimum basilicum,* as Antifeedants Against Larvae of the Gypsy Moth, *Lymantria dispar*

**DOI:** 10.1673/031.013.16101

**Published:** 2013-12-30

**Authors:** Zorica Popović, Miroslav Kostić, Sladjan Stanković, Slobodan Milanović, Ivan Sivčev, Igor Kostić, Petar Kljajić

**Affiliations:** 1 Department of Ecology, Institute for Biological Research, University of Belgrade, Bulevar despota Stefana 142, 11060 Belgrade, Republic of Serbia; 2 Institute for Medicinal Plant Research “Dr Josif Pančić,” Tadeuša Košćuška, Belgrade, Republic of Serbia; 3 Institute for Science Application in Agriculture, Bulevar despota Stefana 68b, 11060 Belgrade, Republic of Serbia; 4 University of Belgrade, Faculty of Forestry, Kneza Višeslava 1, 11030 Belgrade, Republic of Serbia; 5 Department of Forest Protection and Wildlife Management, Faculty of Forestry and Wood Technology, Mendel University, Zemědělská 3, 613 00 Brno, Czech Republic; 6 Institute for Plant Protection and Environment, Banatska 33, 11080 Zemun, Republic of Serbia; 7 Institute for Multidisciplinary Research, Kneza Višeslava 1, 11030 Belgrade, Republic of Serbia; 8 Institute for Pesticides and Environmental Protection, Banatska 31b, 11080 Zemun, Republic of Serbia

**Keywords:** botanical antifeedant agents, chlorophyll fluorescence

## Abstract

Ethanol solutions of five fractions obtained from essential oil of sweet basil *Ocimum basilicum* L. (Lamiales: Lamiaceae) (F1–F5) were tested for their antifeedant properties against 2^nd^ instar gypsy moth larvae, *Lymantria dispar* L. (Lepidoptera: Lymantriidae), in laboratory non-choice and feeding-choice experiments. Prior to bioassays, the chemical composition of each fraction was determined by gas chromatography analyses. Significant larval deterrence from feeding was achieved by application of tested solutions to fresh leaves of the host plant. The most effective were were F1 (0.5%), F4 (0.05, 0.1, and 0.5%), and F5 (0.1 and 0.5%), which provided an antifeedant index > 80% after five days. A low rate of larval mortality was observed in no-choice bioassay. In situ screening of chlorophyll fluorescence as an indicator of plant stress level (assessed by the induced fluorometry) confirmed that the tested compounds did not cause alternations in the photosynthetic efficiency of treated leaves.

## Introduction

Gypsy moth, *Lymantria dispar* L. (Lepidoptera: Lymantriidae), caterpillars are major defoliators of deciduous forests and urban environment (i.e., orchards, parks, and tree rows). Along with conventional treatments that are implemented in forest management and protection, biological means of regulation of *L. dispar* population density were incorporated in the integrative management concept (based on *Bacillus thuringiensis)* (Flexner 1986; Sample 1996).

Due to concern about the environment, there is a need to devise new means of protection that are of natural origin and without consequences for ecosystem functioning. The most desirable product should be non-lethal even to target species, but able to repel the pest. Essential oils, which can be obtained from fresh or dried plant material through simple non-expensive procedures, consist of secondary metabolites (commonly a mixture of monoterpenes, sesquiterpenes, phenols). In comparison with chemically synthesized products, they are distinguished by minimal adverse effects to human and environmental health, with significant biological activity as antimicrobial, antifungal, antitumor, insecticidal, and herbicidal agents (Reigosa et al. 2001; Isman 2004). One of the well-known natural bio-pesticide sources to protect cultivated plants from insects is the neem tree, *Azadirachta indica* Jussieu (Sapindales: Meliaceae), containing azadirachtin with insecticidal and strong repellent properties. The commercial formulation of azadirachtin, Bioneem, is widely used and highlighted for its antifeedant properties. According to Chapman (1974), antifeedants are specific semiochemicals derived as plant secondary metabolites, which are active in the last step of animal-host selection behavior, limiting the diet for both larvae and adult insects.

New products of natural origin that could be used in plant protective programs have been well-studied in recent years, but little has been done to evaluate the effects of these new products on the treated plant. Therefore, this positive tendency of promoting the natural products for plant protection should be supported with this kind of research. This study was undertaken to evaluate antifeedant activities of five fractions obtained from essential oil of sweet basil, *Ocimum basilicum* L. (Lamiales: Lamiaceae), to *L. dispar* larvae. The selection of these compounds for study was indicated by several authors who investigated sweet basil essential oil for its insecticidal activities (Umerie et al. 1998; Asian et al. 2004; Popović et al. 2006; Murugan et al. 2007). Marković et al. (1996) and Kostić et al. (2008) clearly showed significant effects of linalool (the main component of sweet basil essential oil) to gypsy moth survival and feeding. This study was expanded to assess the influence of tested compounds to the photosynthetic performance of the treated plant.

## Materials and Methods

### Plant material

*Ocimum basilicum* plants were collected from Pančevo, Serbia. Specimens were deposited at the Institute for Medicinal Plant Research, Belgrade. Fresh leaves of *O. basilicum* were harvested during the flowering period. The leaves were air-dried at room temperature (22–25° C) for 7 days. To obtain the essential oil from dried leaves, a Clevenger-type apparatus was used, in accordance with standards of Yugoslav (Federal Bureau of Health Care 1984) and European (European Directorate for the quality of Medicines 2002) pharmacopeias. Five fractions (F1–F5) were isolated from this essential oil by low resolution vacuum refraction, transferred into dark glass flasks filled to the top, and kept at temperature of 4° C until used.

### Chemical characterization of obtained fractions

The composition of the examined fractions was determined by gas chromatography (GC) and mass spectra (MS) analyses, as described by Block et al. (2006). GC analyses were performed using an HP-5890 Series II gas Chromatograph (www.hp.com) with a split/splitless injector, fused silica capillary column (25 m × 0.32 mm) coated with non-polar stationary phase HP-1 (cross-linked methylsilicone, 0.5 μm film thickness), and a flame ionization detector. GC/MS analyses were done on a Hewlett-Packard 5890 gas Chromatograph directly coupled to a Hewlett-Packard HP 5971 A (70 eV) mass selective detector. Component identification in tested samples was carried out by GCD ChemStation Software G1701BA version B.00.00 (Agilent Technologies, www.chem.agilent.com) using the probability merge search engine along with Wiley 275 L. mass spectral database library (www.onlinelibrary.wiley.com), comparing MS of recorded constituents with those from the Agilent MS library.

### Preparation of test solutions

Prior to bioassay, each fraction was serially diluted with 96% ethanol to prepare test solutions of 0.05%, 0.10%, and 0.50%.

### Botanical insecticide standard

Bioneem (0.09% azadirachtin, Safer, www.saferbrand.com) was used as the botanical standard control in all experiments (Zabel et al. 2002). The commercial preparation was diluted with 96% ethanol to prepare test solutions of 0.05%, 0.10%, and 0.50%.

### *Lymantria dispar* culture

*Lymantria dispar* egg masses from natural populations were collected from National Park Djerdap (Eastern Serbia) during the autumn period, and egg masses were maintained at 4° C until the next spring. Prior to bioassays, eggs were mechanically cleaned and disinfected (dipped in 0.1% Na-hypochloride for 5 min), then washed with distilled water for 10 min and air-dried. Vital eggs from 25 egg masses were intermixed and put into flasks for hatching (at 25° C). Newly hatched larvae were selected and maintained together in Petri dishes (diameter = 20 cm) until they reached the 2^nd^ larval stage. Caterpillars were daily nourished with fresh leaves of cherry plum, *Prunus cerasifera* Ehrh. (Rosales: Rosaceae). Caterpillars were maintained, and all experiments were carried out, in a microclimate chamber at 25 ± 1° C, 65 ± 5 % RH, and neon diffuse light with 30159.29 candelas with a 16:8 L:D photoperiod (Odel et al. 1985).

### Antifeedant activity of botanical preparations

**Figure 1.**
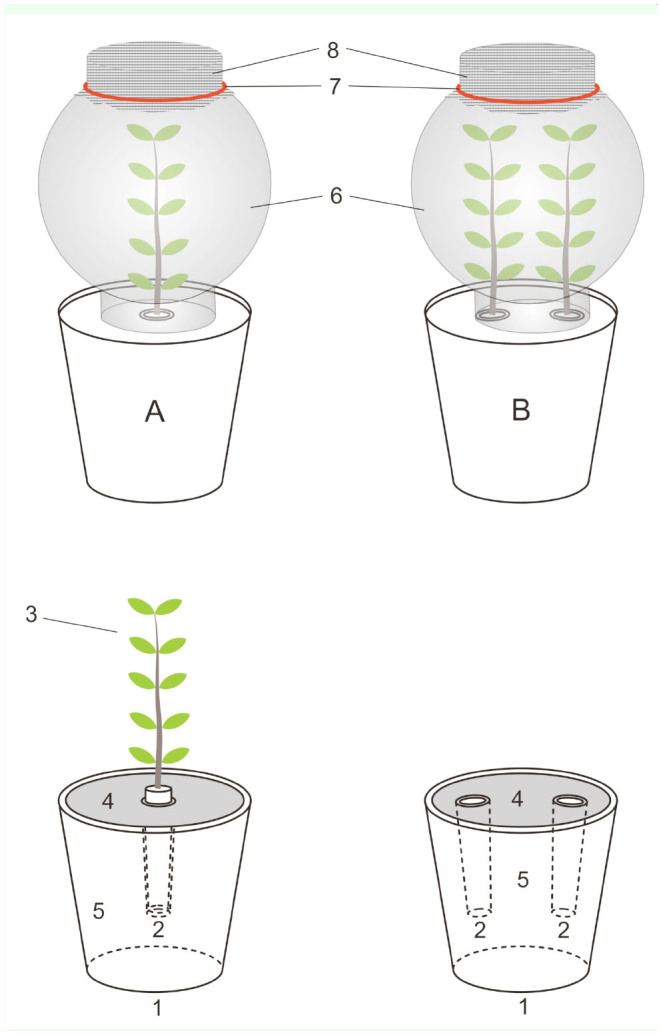
Experimental design for non-choice (A) and feeding choice (B) assays of *Lymantria dispar.* Schematic presentation of experimental setup where 10 larvae were introduced under glass cylinder: branches of *Prunus cerasifolia* with 10 leaves (3) were put into flasks with water (2) and then held in the pots (1) with sand (5) covered with filter paper on the top (4); leaf mass was treated by spraying with test solutions, and after the drying of deposit, glass cylinders (6) covered with cheesecloth (8) fixed with rubber (7) were put for isolation of the treatment. In the no-choice feeding assay, a branch was treated with tested solutions (A), while in the choice feeding assay, one branch was treated with tested solutions and another remained untreated (B). High quality figures are available online.

**No-choice feeding assay.** For this investigation, small branches of *P. cerasifolia* (20 cm long with uniform leaf mass) were used. Branches were put into flasks with water and then held in the pots with sand. Leaf mass was treated by spraying it with each tested solution with a TLC sprayer (Sigma-Aldrich, http://www.sigmaaldrich.com) where the solution deposit was 3.0 ± 0.3 mg/cm^2^, e.g., a total of 40 mL of solution per m^2^ was used for the treatments. When the deposit dried (about 20 min), glass cylinders were put on for the isolation of the treatment and larvae (Figure 1). Then, 10 larvae per replication were introduced. Experiments with both the botanical standard control (in three concentrations: 0.05%, 0.10%, and 0.50%) and the technical control (ethanol 96%) were performed under the same conditions. Dead larvae were counted and removed after 24, 72, and 120 hr. The treatments were replicated six times (total: n = 60). Mortality was expressed in percentages.

**Feeding choice assay.** For feeding choice test, two branches of *P. cerasifolia* (both the same leaf mass, the same age, and from the same tree) per treatment were used. Leaves of one branch were treated by spraying with test solution using a TLC sprayer where the solution deposit was 3.0 ± 0.3 mg/cm^2^, and the other branch remained untreated. After the deposit dried (about 20 min), both branches were put into flasks with water and fixed into the pots with sand. Then, 10 larvae per replication were introduced, and glass cylinders were put to isolate each treatment. Experiments with both the botanical standard control (in three concentrations: 0.05%, 0.10%, and 0.50%) and the technical control (ethanol 96%) were performed under the same conditions. The treatments were replicated six times (total: n = 60).

Prior to bioassay, the average leaf area of *P. cerasifolia* leaves was determined by scanning 30 leaves and calculating the average leaf area in the software package Image Tool (http://compdent.uthscsa.edu/dig/itdesc.html). Each branch had 10 leaves with an average area of 963 ± 9.75 mm^2^.

The antifeedant index (AF) was calculated on the basis of percent area eaten on treated and control leaves using the equation:


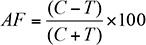


where C is the area (mm^2^) of the control leaf consumed, and T is the area (mm^2^) of the treated leaf consumed (Simmonds et al. 1989). Applying this formula, AF could be in the range from -100 to +100; if a value of AF > 0 was obtained, the compound was considered to be an antifeedant. Leaf area eaten was visually estimated after 24, 48, 72, 96, and 120 hr on a scale of 0 to 100%. This value was transformed into a consumed area expressed in mm^2^ ((leaf area eaten/100)*963mm^2^*10, where 963 is the average area of one leaf and 10 is the number of leaves on branch).

### Phytotoxic activity of fractions: Estimation of photosynthetic activity

Chlorophyll fluorescence measurements were performed in a greenhouse on two-year-old seedlings of *P. cerasifolia*, which were transferred from the nursery and transplanted into 15-L containers filled with a substrate of local soil, sand, and farm yard manure (volume proportion 2:2:1). From March to July, when measurements were undertaken, the glasshouse conditions were as following: air temperature 25–37° C, RH around 54%, natural photoperiod, and uniform watering twice a day. The experiment was settled in three repetitions as follows: each fraction (F1–F5) was applied on nine seedlings (three for each concentration), botanical standard control was applied on nine seedlings (three for each of concentrations), and alcohol control was applied on three seedlings (total number of plants: n = 57). The compounds were applied using the same sprayer and amounts as in feeding assays. Steady state fluorescence was determined with a plant stress meter (Biomonitor, www.biomonitor.dk) by the method of induced fluorometry (Powels 1984; Oquist and Wass 1988). The photosynthetic function was assessed by the rate of basic fluorescence, i.e., the ratio of variable to maximal fluorescence (F_v_ / F_m_ = F_m_ - F_o_ / F_m_, where F_o_ and F_m_ are initial and maximal fluorescence of darkadapted leaves). Each leaf was illuminated with saturating low light (100 μmolm^2^s^-1^) for 2 sec after having been in darkness for at least 20 min. Measurements were performed at 24, 72, and 120 hr after the application of solutions.

### Statistical analysis

Data analysis included the calculation of the mean values and the analysis of variance, where different concentrations of tested solutions, alcohol and control (untreated), were independent variables. For data on larval mortality in no-choice feeding tests, analyses of variance was performed with arcsine transformed data and presented in percentages. AF index was calculated according to the given formula (with arcsine transformed data) and expressed in percentages. Differences between mean values in each set of data were additionally tested by Duncan's multiple range test at the 5% level.

Differences among mean values of F_v_/F_m_ were taken as significant if *p* < 0.05, tested by one way break-down ANOVA test.

## Results

### Component analysis of fractions of essential oil of *Ocimum basilicum*

GC-MS analysis of essential oil of *O. basilicum* detected a total of 37 components. Of these, 20 compounds were present in F1, 21 in F2 and F3, 24 in F4, and 30 in F5. Linalool was the dominant component in all fractions. Principal differences in the chemical composition of separated fractions were as follows: (1) F1 contained limonene and 1,8-cineole; (2) F2–F4 contained mostly similar compounds, with linalool being the most dominant (> 90%), followed by estragole (2.5–3.5%) and other compounds present in low concentrations; (3) F5 contained a group of several compounds (approximately 35% of its total components) that were mostly missed, or were detected in minor concentrations, in other fractions (for example, b-selinene, a-selinene, g-quanene, d-cardinene, a-cadinol). Strong volatiles, including geraniole, eugenole, and b-caryophyllene, were more than 20 times more present in F5 than in the others.

### Non-choice feeding assay (larval mortality)

After 24 hr of larval exposure to tested compounds, mortality was 2.5–42.5%. F2 applied in both concentrations (0.1% and 0.5%) caused the highest mortality of larvae. The dynamics of larval mortality during the time of observation is shown in Table 1. Initial toxicity was not exhibited by tested solutions, with the exception of F2 activity.

### Choice feeding assay (AF index)

After the first 24 hr of the feeding choice experiment, the highest values of AF index were noted on leaves treated with F4 and F5 (Table 2). F1 and F2 at a concentration of 0.05% exhibited phagostimulant activity, but only in the first evaluation. F2 showed much stronger phagostimulant activity in comparison to Fl. During the experiment, F4 and F5 treatments retained their antifeedant properties, i.e., the highest AF index (> 80%) was noted on leaves treated with these fractions. F1, F2, and F3 were also effective in protecting leaves from larval feeding. Their AF index was 65–75% and lasted throughout the 5 days of observation.

### Photosynthetic activity of leaves treated with examined compounds

The results of photosynthetic efficiency (expressed as F_v_/F_m_ value) of plants treated with the tested compounds are presented in Table 3. The fluorescence parameters of leaves treated with fractions after 5 days did not show any differences compared to the control. In leaves treated with fractions F4 and F5 (at all three concentrations), values of F_v_/F_m_ were stable from the beginning of the experiment, and no significant differences compared to the control were observed. Treatment of leaves with fractions F1, F2, and F3 caused a decrease in photosynthetic efficiency within the first 72 hr, but then values became stable and didn't differ significantly from the control. Leaves treated with F2 (0.05%) and F3 (0.50%) had significantly lower F_v_/F_m_ from the first observation (24 hr) and throughout the experiment. Since these values didn't have common dynamics, depending on concentrations and observation time, the decrease of F_V_/F_m_ values could be attributed to weaker adaptation of plants and possible damage during transplantation. The results showed significant differences in F_v_/F_m_ values for leaves treated with Bioneem (all three concentrations) compared to the control.

## Discussion

Some previously reported findings indicate that secondary plant metabolites obtained from *O. basilicum* exhibited insecticidal and repellent activities (Umerie et al. 1998; Grayer 1999; Zabel et al. 2002; Popović et al. 2006; Kostić et al. 2008). It is widely accepted, and shown in our study, that essential oils are generally of low acute toxicity (Schumutterer 1985), and that highly complex mixtures of terpenoids, particularly monoterpenoids and related phenols in essential oils, jointly contribute to repellency and feeding deterrence (Isman 2006).

All fractions tested in our study caused a significant decrease of larval feeding in 2^nd^ instar larvae in the bioassay. Since the larval feeding was inhibited after the application of tested compounds, it could be concluded that these fractions exhibited primary (or gustatory) antifeeding activity, which is “the inability to ingest resulting from the perception of the antifeedant at a sensory level” (Schumutterer 1985), following the same principle of action as Bioneem (Mordue and Nisbet 2000).

Plant damage caused by insect feeding is initiated by release of plant volatiles (attractants and phagostimulants) that can help herbivores locate their hosts (Pare and Tumlinson 1996). The results of our study suggest that fractions of *O. basilicum* essential oil and Bioneem sufficiently inhibited the responses of larvae to these specific stimuli, even in low concentrations. The physical properties of the tested solutions probably were not significant in the sense of feeding inhibition, since there were no visible differences between treated and untreated leaves. Therefore, prevention of leaf damage by feeding achieved by the application of tested compounds could be mainly attributed to their volatile compounds.

Time dependent antifeedant activity was significant, i.e., protective properties of tested solutions increased during the observation time. Larval desensitization to deterring volatiles did not occur. Moreover, some of larvae that consumed small amounts of treated leaves stopped feeding the next day (the AF index increased daily). The AF index was relatively high for all tested solutions, with small differences in efficiency of fractions depending on their chemical composition. We suggest that strong volatiles, especially in their synergic action, were important for masking the effect of tested solutions on feeding by *L. dispar* larvae (Chapman 1974). Desensitization to antifeedants in feeding assays has been documented for lepidopteran larvae (Bomford and Isman 1996). However, in our experiment the antifeedant index was maintained at a high level during the observation time for all treatments.

Fractionation of the essential oil could indicate the particular group of compounds with biological activity. This analysis confirmed that the biological activity of each fraction was different, although linalool was the dominant component in each fraction, suggesting antifeedant properties of other chemical compounds, perhaps having synergistic activity. High activity of F4 could be the consequence of the strong volatile estragole, which has not been investigated in bio-tests with insects. Fraction F5 was rich in biologically active compounds with proven antifeedant properties, including eugenole (Ravi Kiran et al. 2007; Eriksson et al. 2008) and geraniole (Momin and Nair 2002). Several constituents of this fraction (γ-cadinene, δ-cadinene, α-selinene, β-selinene, α-cadiol) are known to have strong volatile properties that have not been investigated for biological activity against insects. Also, F1 was noted as having strong initial and lasting antifeedant properties, which could be due to the presence of limonene and 1,8-cineole. These compounds have already been confirmed as effective in prevention of feeding of different insects (Tripathi et al. 2001; Shukla et al. 2011; Suthisut et al. 2011), including moth larvae (Riva Kiran et al. 2006; Fan et al. 2011). F2 wouldn't be suitable as a potential protective agent against *L. dispar* larvae, because it showed high initial toxicity and lower AF activity compared to other fractions. Considering the chemical composition of this fraction, its biological activity could be attributed to the highest content of linalool.

Photosystem II functioning is sensitive to a wide range of environmental variations, so the chlorophyll fluorescence provides considerable information on the effects of stress on plants (Schreiber et al. 1994). Testing plant photosynthetic function in this way is widely used for estimation of effects of herbicides, non-herbicidal pesticides, insecticides, and some allelochemicals (Krugh and Miles 1996; Reigosa et al. 2001; Spiers et al. 2006; Bigota et al. 2007).

According to our results, the photosynthetic function of treated *P. cerasifolia* leaves was not negatively affected when tested fractions from *O. basilicum* essential oil were applied. Previous research on changes in photosynthetic efficiency of plants exposed to different products for plant protection reported a decrease in photosynthetic gas exchange, chlorophyll fluorescence (mostly reversible), pigment constitution, and overall growth rate (Krugh and Miles 1996; Reigosa et al. 2001; Spiers et al. 2006; Bigota et al. 2007; Ali and Al-Quraishy 2008; Khillar et al. 2010; Pattisona et al. 2011). Our results suggest that fractions obtained from *O. basilicum* essential oil may hold promise as novel and effective leaf protective agents from *L. dispar* larvae, with no adverse effect on plants or the environment. However, it is necessary to do more testing on a range of plant species, due to differences in morphology, anatomy, and physiology of different taxa, in particular to test the sensitivity of certain crops and genetically modified species.

## Conclusion

*O. basilicum* essential oil was separated into fractions, and five natural products of specific chemical composition were obtained. Each fraction was characterized as being complex mixtures of volatile compounds, which were used in further bioassays. All tested fractions had low toxic effects, but had significant and lasting antifeedant properties to 2^nd^ instar *L. dispar* larvae (with the exception of F2, which showed high toxicity and a lower AF effect). Estimation of photosynthetic efficiency of leaves treated with tested fractions showed no adverse effect on the photosynthetic performance of plants after the application of fractions. The study of these effective products with no adverse effects on plant productivity and overall ecosystem balance needs to be continued and extended to other plant species and phytophagous pests.

**Table 1.**
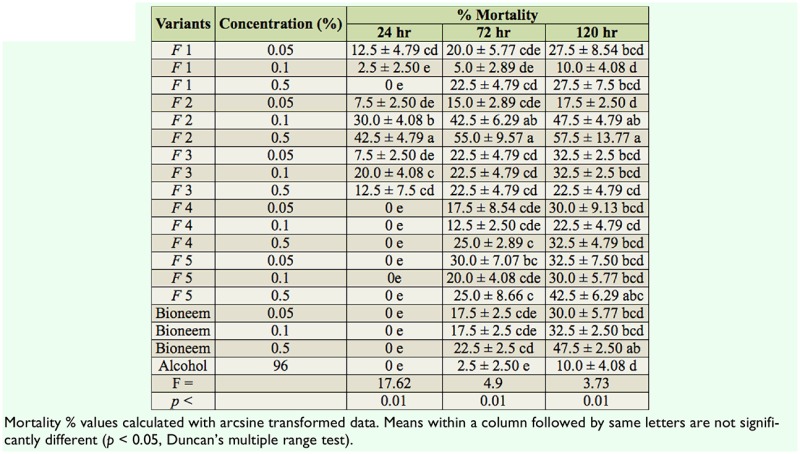
Mortality of *Lymantria dispar* in the non-choice assay during the five days of observation after different hours of larval exposure.

**Table 2.**
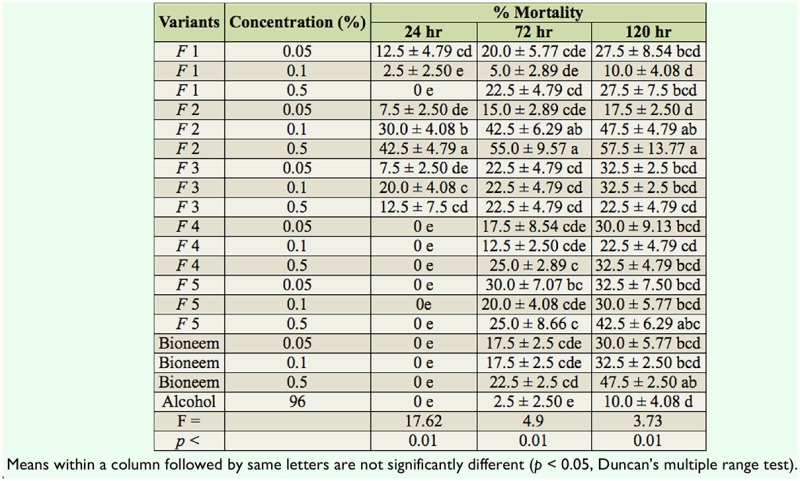
Antifeedant index (%) during the five days of observation after different hours of larval exposure.

**Table 3.**
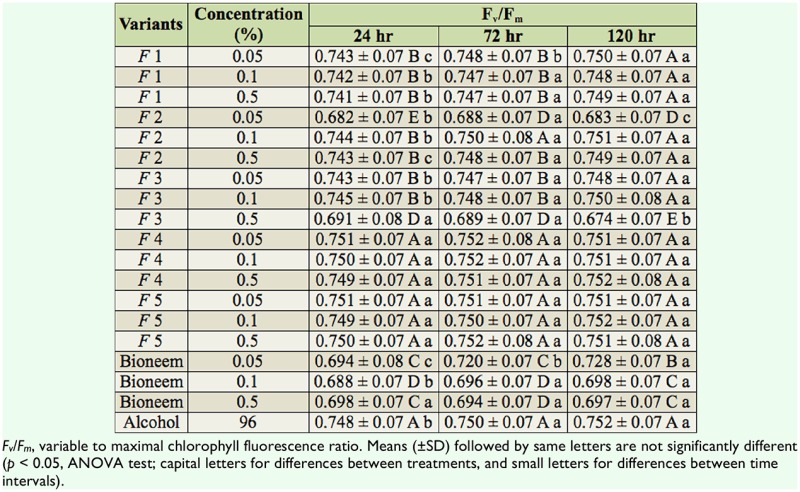
Photosynthetic efficiency (F_v_/F_m_ values) of *Prunus cerasifolia* leaves treated with tested compounds after different times from the application of tested compounds.

## Abbreviations

AF,: antifeedant index;
F1–F5,: five fractions of essential oil;
GC,: gas chromatography;
MS,: mass spectra
